# Supplementary material for: Transcription decouples estrogen-dependent changes in enhancer-promoter contact frequencies and spatial proximity
Source: PLoS Genet. 2024 May 23;20(5):e1011277. doi: 10.1371/journal.pgen.1011277 (PMC11152312; doi:10.1371/journal.pgen.1011277)
Supplement: Attachment — Submitted filename: 138803_1_rebuttal_2843870_s3p7dr.docx [file pgen.1011277.s016.docx]

**Manuscript number:** RC- -2023-02106

**Corresponding author(s):** Wendy Bickmore

## 1. General Statements

We thank the reviewers for their critiques of our manuscript and for recognizing the importance of the questions about 3D genome organisation that it addresses. We plan to address most of their comments in our revised manuscript.

## 2. Description of the planned revisions

*Insert here a point-by-point reply that explains what revisions, additional experimentations and analyses are planned to address the points raised by the referees.*

Reviewer #1

i. *The aneuploid karyotype of the MCF-7 cells used is a concern. GREB1 is present in four copies, with two on abnormal chromosomes which may not be regulated in the same way as primary cells. The authors should include caveats to this effect in the text to account for this*.

We indicated (pg 5) that there are 4 copies of GREB1, 2 of which are on re-arranged chromosomes. RNA FISH (Figure 1C) suggests all 4 of these alleles are induced by estrogen. On each allele, the GREB1 enhancer and promoter remain closely apposed by imaging (Figure 2, DNA FISH) indicating no gross chromosomal rearrangements around the GREB1 locus. This is confirmed by our Hi-C data (Figure 2A), where any genomic rearrangements at the GREB1 locus would be detectable when the sequencing data were aligned to the reference genome. In the revised manuscript we highlight these points in the respective results sections (pgs 5 and 6). Our data suggest that all 4 alleles of GREB1 in MCF7 cells are regulated in the same way.

ii. *The authors should also include more information on the generation and verification of the enhancer deletion cell lines. An illustration of the PCR primers used for screening, as well as an illustration of the sequenced product traces aligned with the reference genome (as opposed to just showing the deleted regions) should be included in Fig. S1D. This would give the reader more confidence that the designed knockout has occurred in the same way on all alleles. Furthermore, long-range PCRs and sequencing should be considered to confirm that no larger deletions have occurred (e.g. Owens et al., 2019 PMID: 31127293).*

We have replaced FigS1D with a new Figure Supplement (Figure S1.2A) that incorporates a more comprehensive diagram of the strategy used for the generation and screening of the enhancer deletion cell lines. This also includes the sequencing traces aligned to the reference genome for each of the clones used in this work. Additionally, in the revised manuscript, we will check the deletions using the C-TALE sequencing data obtained from the enhancer-deleted clones.

iii. *The changes in the measured E-P interaction frequency following gene activation are* ***weak*** *at best and make visual interpretation of the results difficult. Showing the reciprocal virtual 4C plots from the promoter would help to reassure the reader that the observed effect is real*.

We thank the reviewer for this suggestion, and we will now include virtual 4C plots from the *GREB1* and *NRIP1* promoters in our revised manuscript. These will be in figures 2B, 2E, 3C, 4C and in the supplementary figures 2B, 4B, 5C and 6C.

iv. *Furthermore, the precise 3C method used is not clear. The authors repeatedly refer to "Capture-C" (a commonly used 3C-based approach using biotinylated oligos to pull down targets of interest) but the citation used (Golov et al.* *2019) refers to a conceptually similar method called "C-TALE". This should be clarified in the text.*

We thank the reviewer for pointing out this potential confusion. We replace the term Capture-C with C-TALE throughout the revised manuscript.

v. *As for the changes in contact frequency, the observed changes in distance measurements between conditions are very small (although statistically significant). We acknowledge that this is likely due to the relatively small linear distances between enhancers and promoters in this study. However, it would be helpful to see the effects of the induction/treatments on a one or more control loci which is not affected by oestrogen signalling given that global changes in nuclear shape/volume and/or cell cycle effects could occur within this time (e.g. effects of tamoxifen treatment on MCF-7 cell cycle distribution, (Osborne et al. 1983 PMID: 6861130), which could impact nuclear volume*.

Data from DNA FISH control probes are already included in Supplementary Figure S3 showing no change in intra-nuclear distances and thus no general effects on chromatin compaction due to nuclear volume or cell cycle. Virtual 4C data for the entire captured regions around *GREB1and* *NRIP1* are show in Fig S2C, also showing no general effect on the wider capture windows. We will include similar data from the viewpoint of the gene promoters in the revised manuscript. Hi-C and imaging data from the enhancer deletion cell lines (Fig S4) also supports that we are looking at an ER-specific effect, not a global one. With the regard to the comment on the effects of tamoxifen treatment on MCF-7 cell cycle distribution, we see no effects of tamoxifen on 3D genome organisation at *GREB1* and *NRIP1* by Hi-C or by imaging.

vi. *The authors discuss previous studies demonstrating that E2 and 4OH recruit different sets of proteins to their target genes. Given that this is central to the conclusion that the ER ligand (and its recruited co-factors) determines the E-P interaction frequency and 3D distances observed, it would be important to demonstrate this at the GREB1/NRIP1 loci specifically. ChIP data of the co-activators/repressors recruited by E2 and 4OH, respectively, would greatly strengthen this claim.*

We acknowledge that investigating co-activator and co-repressor recruitment to the studied loci will strengthen our interpretation our conclusions. In the revision we will perform and include ChIP-qPCR at *NRIP1*, *GREB1* and control loci assaying for PolII, co-activators such as p300, mediator and SRC-3 and the co-repressors N-CoR in control, estradiol and tamoxifen treated cells. We will also perform ChIP-qPCR of PolII and co-activators in cell treated with flavopiridol and triptolide.

vii. *The observed uncoupling of E-P contact frequency and 3D distance upon transcriptional inhibition is interesting and offers clues to the molecular details underlying E-P interactions. However, the use of flavopiridol and triptolide, while common in the field, should be carefully qualified given the potential for their indirect effects on transcription. This is particularly important for flavopiridol given its ability to target multiple cyclin-dependent kinases beyond CDK9 and its role in transcription initiation*.

In the revised manuscript we indicate that “Flavopiridol inhibits several CDKs, including CDK9/PTEF-b”

Minor comments:

viii*. In the introduction and beginning of discussion, it would be helpful to detail previous studies where FISH-based analyses have shown more proximal E-P positioning upon activation, to make it clear that differences in E-P proximity appear to be gene-specific. Some examples include Williamson et al. (2016; PMID 27402708) and Chen et al. (2018; PMID 30038397). Speculation as to why some genes behave in this way while others do not, would also be worthwhile*.

We have followed the reviewer’s suggestion and noted these two studies in the Introduction of a revised manuscript. Given that the focus of this current manuscript is to explore discrepancies between Hi-C and DNA FISH, we do not think that this is the right forum for a wider discussion of why there might be differences in E-P proximity between different biological systems.

ix. *On page 6, the authors state that after deletion of the NRIP1 enhancer there is "almost total loss of NRIP1 induction in response to E2". This does not seem to match the data where in 3 out of 4 replicates (2 for each clone) there is a statistically significant increase in number of RNA FISH foci upon E2 stimulation in the NRIP1 enhancer KOs. This suggests that, as for GREB1, the regulation of these genes is not solely controlled by the deleted enhancers. This should be clarified in the text.*

The reviewer is referring to the data on *NRIP1* expression in two NRIP1 enhancer deletion clones in Fig 1D and the replicate data in Supplementary Fig S1 (upper-right panel). These data show almost no induction of NRIP12 by E2 compared to wild-type cells. We stand by our statement.

x. *The labelling of the FISH probes in Supp. Fig. S2 could be improved as it is currently very difficult to read these*.

We will try to improve this in a revised Figure S2.

xi *Given that the authors have referenced a distance of 200 nm as potentially being an important threshold for gene activation, it would be useful to include the fraction of alleles which are below this distance alongside the cumulative frequency plots in Figure 2D and elsewhere in the paper as the cumulative frequency plots can be hard to read in some cases (e.g. Supp. Fig. S3B e-p). This would also allow the authors to show consistency across replicates*.

We thank the reviewer for this suggestion to make the data easier to interpret. In a revised manuscript, we will incorporate the fraction of alleles below and above 200 nm for the DNA-FISH experiments in Figure 2D and Figure S4A-B.

xii. *For clarity, it would be helpful to include the difference map between the vehicle-treated unstimulated/stimulated conditions for the 3C plots in Fig. 4. This would help contextualise the resulting differences observed with the drug treatments. Same for Supp. Fig. S6*.

We will include the difference heatmap between the vehicle- and estradiol treated samples for vehicle, flavopiridol and triptolide treated samples.

xiii. *Statistical comparisons are not shown for all 3D FISH-based distance measurements (e.g. Supp. Figs. S3A, S4C, D, S6E). If this is because the tests were done and the results were non-significant this should be indicated*.

We had omitted all non-significant p values (>0.05) from the graphs to stop them getting too cluttered. All p values are documented in the supplementary tables. However, following the reviewer’s comment, we will indicate all non-significant statistical comparisons on the graphs.

xiv. *On page 13, the authors state that increased E-P separation occurs "before nascent transcription of the gene is detected by either TT-seq or RNA FISH". This does not appear to be correct given that baseline levels of transcription are observed in the absence of ER stimulation by both methods (Fig. 1). This should be clarified in the text.*

We have amended this statement to now indicate that “This is before an induction of nascent transcription of the gene….”

Reviewer #2

i. *The authors make strong claims and although these are generally reasonably well supported by the data, it is important to acknowledge that they are based on two loci. This manuscript would be stronger if the authors could include additional loci in their study design. If this is not possible, it would be good to acknowledge that the conclusions are preliminary/speculative at this stage*.

The reviewer makes a fair point, and we emphasized throughout the text – including at the end of the Discussion - that we are examining just two gene loci. In a revised manuscript we will include DNA-FISH data for a third locus comprising the *CCND1* gene, for which we have preliminary data.

ii. *It would be helpful if the authors could clarify the strategy they used for their FISH probe design. The enhancer and promoter fosmid probes (which are used for the majority of the experiments) are not centered on the active elements and do not even seem to overlap in the case of the GREB1 enhancer fosmid probe. The 10 kb enhancer probe seems better placed for the GREB1 locus, but the 10 kb enhancer probe does not seem to overlap with the enhancer in the NRIP1 locus. It is conceivable that the exact location of the probes has a big impact on the measurements and it would therefore be helpful if the authors could comment on the location of the probes and add additional probes if required to strengthen their conclusions. In addition, the fosmid probes are very large (40 kb). Although the authors acknowledge this, it would be helpful if they could comment on how overlap between 40 kb probes should be interpreted in relation to a potential rather focal contact between (proteins bound to) regions of <1 kb.*

In the case of GREB1, the fosmid probes were chosen to maximize the distance between them as the promoter and the enhancer of the gene are genomically relatively close to each other. This was not an issue in the case of the NRIP1 locus where fosmid probes could be placed centered on the TSS and the enhancer region. In the case of the 10 kb probes, these were designed to be centered on the regions where higher E2-induced C-TALE contact frequencies were detected. Virtual 4C plots using the TSS regions as viewpoints (incorporated into the revised manuscript) clearly show that, in the case of NRIP1, the contact frequency peak does not fall on the main ER peak.

iii. *It is not clear to me why the authors would choose to work with a locus that is present in 4 copies in their cell line. Is the entire regulatory region (incl. enhancers) preserved for the two additional copies of the gene? Can the authors comment on how this may impact on their measurements?*

See response to Reviewer 1, point 1. Our Hi-C data would have revealed if there were genomic rearrangements in the 600kb window surrounding *GREB1*.

iv. *Figure 2D shows an increase in E-P separation for the NRIP1 locus across all timepoints, with cumulative frequency plots shown for the 10 min timepoint. However, the data for the second replicate shown in Figure S2D are a lot less robust and not significant for the 10 min timepoint. It is important that the authors either provide additional data to support the robustness of this experiment or acknowledge that the results are not fully reproducible.*

We acknowledge this, but we would like to note that there is an increase in the median distance for all time points, although this difference is not significant in some of the timepoints. Additionally, DNA-FISH data obtained using the 10 kb probes confirm these observations.

v. *The data presented in Figure 2F for clone 2 of the GREB1 enhancer deletion still show increased E-P distance upon activation. How do the authors explain this*?

This increase in distance is not statistically significant (p-0.33 – see Table S2) and is not seen for the replicate data in Fig. S4.

Minor comments:

vi. *Could the authors comment on the observation that the NRIP1 promoter is not bound by ERa or p300 upon estrogen activation? Are there ATAC-seq or H3K27ac ChIP-seq data available for these conditions?*

We included ATAC-seq tracks in Figure 1A where a peak on the *NRIP1* promoter is clearly seen.

*vii. It* *is not obvious which timepoint is shown in Figure 1D.*

Pre-mRNA FISH in enhancer deleted clones was done in cells treated with vehicle or E2 for 60 minutes. This will be made clearer in the figure legend.

viii. *Why did the authors choose e-i and p-i instead of e-c and p-c in Supplementary Figure 3B?*

We apologize as it was an oversight not to include the e-c data for this experiment. This is now included in Supplementary figure S4B.

ix. "*We treated hormone starved MCF-7 cells with flavopiridol or triptolide for 5 min before adding E2 for 30 min (Fig. 4A)." Does this mean that the FLV/TRP treatment lasted for 35 min or did the authors wash it out before adding E2? Please clarify*.

This observation is correct, and it was made clear in Figure 4A and in the figure legend.

x. *The authors refer to their Capture-C data as "high-resolution". However, the methods section mentions that the data for the GREB1 and NRIP1 locus are 5 kb and 10 kb resolution, respectively. This is not particularly high for a targeted approach, certainly not in light of the MNase-based approaches that have recently been developed. I therefore think that the "high-resolution" claims should be removed from the paper*.

In line with the reviewer’s suggestion, we have removed the term high-resolution when referring from our own data.

## 3. Description of the revisions that have already been incorporated in the transferred manuscript

*Please insert a point-by-point reply describing the revisions that were already carried out and included in the transferred manuscript. If no revisions have been carried out yet, please leave this section empty.*

1. “Capture-C” has been replaced by “C-TALE” throughout.

2. We have included ATAC-seq tracks in Figure 1A where a peak on the *NRIP1* promoter is clearly seen.

3. We have replaced FigS1D with a new Figure Supplement (Figure S1.2A) that incorporates a more comprehensive diagram of the strategy used for the generation and screening of the enhancer deletion cell lines. This also includes the sequencing traces aligned to the reference genome for each of the clones used in this work.

4. We now include virtual 4C plots from the *GREB1* and *NRIP1* promoters in figures 2B, 2E, 3C, 4C and in the supplementary figures 2B, 4B, 5C and 6C.

5, We now indicate that “Flavopiridol inhibits several CDKs, including CDK9/PTEF-b”

6. We have added the fraction of alleles < and > 200 nm for the DNA-FISH experiments in Figure 2D and Figure S4A-B.

7. We include e-c data in Supplementary figure S4B.

6. We now indicate all non-significant statistical comparisons on the graphs.

## 4. Description of analyses that authors prefer not to carry out

*Please include a point-by-point response explaining why some of the requested data or additional analyses might not be necessary or cannot be provided within the scope of a revision. This can be due to time or resource limitations or in case of disagreement about the necessity of such additional data given the scope of the study. Please leave empty if not applicable.*
