# Supplementary material for: Transcription decouples estrogen-dependent changes in enhancer-promoter contact frequencies and spatial proximity
Source: PLoS Genet. 2024 May 23;20(5):e1011277. doi: 10.1371/journal.pgen.1011277 (PMC11152312; doi:10.1371/journal.pgen.1011277)
Supplement: Attachment — Submitted filename: PLoS Genetics.docx [file pgen.1011277.s018.docx]

**Transcription decouples estrogen-dependent changes in enhancer- promoter contact frequencies and spatial proximity**

Reviewer #2

Both reviewers shared similar concerns about the conclusions of this work. Based on the comments from both reviewers, the authors have made appropriate revisions to their manuscript and pointed out some of the caveats in their interpretation. The overall conclusion is reasonable: “Our results emphasize that the relationship between contact frequencies and physical distance in the nucleus, especially over short genomic distances, is not always a simple one”. It will be of interest to see the extent to which these observations are shared at other loci. As pointed in a recent review (Yang and Hansen, Nature reviews 2024), reconciling the relationships between chromosome conformation and imaging data is fraught with technical problems. In addition, the data presented here are not clear cut as discussed in the original reviews. It might be worthwhile to include a couple of sentences about this review and add in the reference.

Two other recent publications relevant to the topics discussed here might also be included (reviewed in Ibrahim, Nat Genetics 2024).

In conclusion, in my opinion, the work as now presented could be published to enable further discussion about the points that have been raised and to encourage others to address these important issues to establish a consensus from further observations.
